# How do countries select and use digital global goods in emergency settings? Lessons learned from the DHIS2 COVID-19 data management experiences in Burkina Faso, Mali and Suriname

**DOI:** 10.1093/oodh/oqae003

**Published:** 2024-05-06

**Authors:** Lauren Gilliss, Caitlin Madevu-Matson, Colleen Boyle, Derek Kunaka, Madina Kouyate, Rahim Kebe, Vijay Sewradj, Stephanie Watson-Grant

**Affiliations:** Country Health Information Systems and Data Use Project, JSI Research & Training Institute, Inc., 2733 Crystal Drive, Arlington, Virginia, 22202, USA; Country Health Information Systems and Data Use Project, JSI Research & Training Institute, Inc., 2733 Crystal Drive, Arlington, Virginia, 22202, USA; Country Health Information Systems and Data Use Project, JSI Research & Training Institute, Inc., 2733 Crystal Drive, Arlington, Virginia, 22202, USA; Country Health Information Systems and Data Use Project, JSI Research & Training Institute, Inc., 286 Auriga Street, Waterkloof, Pretoria, 0181, South Africa; Country Health Information Systems and Data Use Project, JSI Research & Training Institute, Inc., Mali Quartier Hamdallaye ACI 2000, Rue, 317, Porte 140, Immeuble SYLLA, Bamako, Mali; Country Health Information Systems and Data Use Project, JSI Research & Training Institute, Inc., Ouaga 2000, Zone A. Section F. Lot 37. Parcelle 03, Ouagadougou, Burkina Faso; Country Health Information Systems and Data Use Project, JSI Research & Training Institute, Inc., Paramaribo, Suriname; Country Health Information Systems and Data Use Project, JSI Research & Training Institute, Inc., 2733 Crystal Drive, Arlington, Virginia, 22202, USA

**Keywords:** COVID-19, digital global goods, digital health, pandemic response, health information systems, DHIS2

## Abstract

Digital health global goods software are accessible, adaptable and scalable tools that can contribute to strengthened health data management and service delivery. This study explored how government stakeholders in three countries (Burkina Faso, Mali and Suriname) selected digital health global goods for COVID-19 vaccine response efforts, including whether and how these digital tools were adapted and scaled, challenges that were encountered and lessons learned from the experience. Primary data were collected through 28 purposively sampled, semi-structured key informant interviews between March and May 2023 with national- and regional-level stakeholders involved in COVID-19 vaccination response efforts. Qualitative data were analyzed deductively and inductively using thematic coding. Ministries of health in each country selected District Health Information Software 2 (DHIS2) as a COVID-19 vaccine tracking information system, but the system selection and configuration process differed. Key factors that most influenced the use of DHIS2 included country familiarity with the tool, urgent demand for real-time data and partner influence. Supportive supervision and reinforcement of data quality contributed to the effective use of DHIS2. Contextual factors, including adequate infrastructure and funding, posed challenges in system use. Experience using DHIS2 during the COVID-19 pandemic has led to plans for its expanded use in other health areas. Based on the findings, the authors recommend ministries of health utilize familiar global goods systems during health emergency periods, donors coordinate to streamline future digital system investments and technical and financial partners support countries to invest in the enabling environment for global goods systems to effectively and sustainably operate.

Abrégé

Les logiciels de produits mondiaux de santé numérique sont des outils accessibles, adaptables et évolutifs qui peuvent contribuer à renforcer la gestion des données de santé et la prestation des services. Cette étude a examiné la façon dont les parties prenantes publiques de trois pays (Burkina Faso, Mali et Suriname) ont sélectionné des produits mondiaux de santé numérique pour des campagnes vaccinales contre la COVID-19, y compris si et en quoi ces outils numériques ont été adaptés et mis à l’échelle, les problèmes rencontrés et les enseignements tirés de l’expérience. Les données primaires ont été recueillies par le biais de 28 entretiens semi-structurés auprès d’informateurs clés échantillonnés de manière ciblée entre les mois de mars et mai 2023 avec des parties prenantes nationales et régionales associées aux campagnes vaccinales contre la COVID-19. Les données qualitatives ont été analysées de manière déductive et inductive à l'aide d'un codage thématique. Les ministères de la Santé de chacun des pays ont choisi District Health information Software 2 (DHIS2) comme système informatisé de suivi de la vaccination contre la COVID-19, mais le processus de sélection et de configuration du système était différent. Les principaux facteurs qui ont le plus influé sur l'utilisation de DHIS2 étaient : la familiarité des pays avec l'outil, la demande urgente de données en temps réel et l'influence des partenaires. La supervision, le soutien et le renforcement de la qualité des données ont contribué à l'utilisation efficace de DHIS2. Des facteurs contextuels, y compris une infrastructure et un financement adaptés, ont présenté des difficultés dans l'utilisation du système. L’expérience de l’utilisation de DHIS2 pendant la pandémie de COVID-19 a conduit à des projets d’extension de son utilisation dans d’autres domaines de la santé. Sur la base des résultats obtenus, les auteurs recommandent que pendant les périodes d’urgence sanitaire, les ministères de la Santé utilisent des systèmes de produits mondiaux connus, que les donateurs coordonnent leurs efforts pour rationaliser les futurs investissements dans les systèmes numériques et que les partenaires techniques et financiers aident les pays à investir dans un environnement propice au fonctionnement efficace et durable des systèmes de produits mondiaux.

Resumen

Los programas informáticos de bienes globales digitales para la salud son herramientas accesibles, adaptables y ampliables que pueden contribuir a reforzar la gestión de los datos sanitarios y la prestación de servicios. En este estudio se examina cómo las partes interesadas gubernamentales de tres países (Burkina Faso, Malí y Surinam) seleccionaron bienes globales digitales de salud para los esfuerzos de vacunación en respuesta a la COVID-19, incluyendo si y cómo estas herramientas digitales fueron adaptadas y ampliadas, los desafíos que se hallaron y las lecciones aprendidas de la experiencia. Los datos primarios se recopilaron a través de 28 entrevistas semiestructuradas a informantes clave con muestreo intencional. Las entrevistas se realizaron entre marzo y mayo de 2023 con partes interesadas a nivel nacional y regional implicadas en los esfuerzos de vacunación contra la COVID-19. Los datos cualitativos se analizaron de forma deductiva e inductiva mediante codificación temática. Los ministerios de Salud de cada país seleccionaron el District Health Information Software 2 (DHIS2) como sistema de información para el seguimiento de las vacunas contra la COVID-19, pero el proceso de selección y configuración del sistema fue diferente. Los factores clave que más influyeron en el uso de DHIS2 fueron la familiaridad del país con la herramienta, la demanda urgente de datos en tiempo real y la influencia de los socios. La supervisión de apoyo y el refuerzo de la calidad de los datos contribuyeron al uso eficaz de DHIS2. Ciertos factores contextuales, como la infraestructura y la disponibilidad de fondos, plantearon retos en el uso del sistema. La experiencia en el uso de DHIS2 durante la pandemia de COVID-19 ha dado lugar a planes para ampliar su uso en otros ámbitos sanitarios. Basándose en los hallazgos, los autores recomiendan a los ministerios de Salud que utilicen sistemas conocidos de bienes globales durante los períodos de emergencia sanitaria; a los donantes, que se coordinen para racionalizar las futuras inversiones en sistemas digitales, y a los socios técnicos y financieros, que apoyen a los países para que inviertan en un entorno propicio en que los sistemas de bienes globales puedan funcionar de manera eficaz y sostenible.

## INTRODUCTION

### Global goods overview

There is limited research on how low- and middle-income countries (LMICs) used existing digital global goods or developed new ones to manage their COVID-19 emergency response. The United States Agency for International Development (USAID) COVID-19 Vaccine Digital Collaborative Learning Agenda defines global goods in the following manner drawing on available literature:

‘Global goods are tools that are adaptable to different countries and contexts to help address key health system challenges. There are three types of global goods in the health sector: (1) content (resources, toolkits or data standards), (2) software and (3) services. They are open-source, adaptable and reusable to meet the diverging needs of various geographic, thematic contexts and digital maturity levels. Mature global goods are free and open source, supported by a strong community, used across multiple countries, have demonstrated effectiveness, are designed to be interoperable and are emergent standard applications’. [1]

While many countries have digital health information systems (HISs), the fragmentation of digital tools and the data they generate have challenged governments in LMICs to effectively respond to pandemics [2,3]. Global goods can help to address this fragmentation by promoting standardization, interoperability, collaboration among stakeholders and open access [4]. Digital global goods are designed to be trusted and replicable tools that can accelerate the scale-up of successful digital health systems and solutions across countries and contexts, rather than creating new tools [4,5]. Global goods advance a safer, more equitable world in the digital era and are promoted in global health strategy documents, including the World Health Organization’s (WHO) *Global Strategy on Digital Health 2020–2025* [6], the United Nations Secretary-General’s *Roadmap for Digital Cooperation* [7], and USAID’s *Vision for Action in Digital Health 2020–2024* [8].

This study focused on the software component of global goods, with District Health Information Software 2 (DHIS2) as a use case given its implementation in the study countries. DHIS2 is an open-source, web-based platform classified as a mature global good that is in use across more than 100 countries worldwide—of which at least 80 are considered LMICs [4,9]. DHIS2 is most frequently used as a health management information system (HMIS) for aggregate, routine reporting of health data using an application within the DHIS2 platform that this paper refers to as ‘aggregate’. Tracker is another application within the DHIS2 platform that enables the collection of longitudinal, individual-level data. Tracker also offers mobile (DHIS2 Capture) and offline capabilities [9]. Each installation of DHIS2 on a server is called an instance and multiple applications like aggregate and Tracker can be used within one instance. The common terminology used to describe the process of setting up or revising DHIS2 is called configuration or customization, and the components that define the structure of the data in DHIS2 are called metadata. Internationally available standardized metadata packages (available at https://dhis2.org/who/) provide pre-configured forms and processes within DHIS2 that enable countries to quickly customize the system based on their needs and context. Linking and comparing data from different applications within one instance of DHIS2 is possible, but requires configuration, such as displaying aggregate data on a dashboard from both aggregate and Tracker applications, or linking individual-level data between different Tracker forms. The integration or interoperability (automated data exchange) between different DHIS2 instances and between DHIS2 and other software applications is common using connectors. In many countries, multiple DHIS2 instances are used, with some serving as the main data collection tool for certain health programs and others serving as a national data warehouse for data visualization and cross-program data analysis.

### COVID-19 response

A variety of global goods were employed for countries’ COVID-19 response efforts because they are open-source, freely accessible, adaptable and standards-based [4,10]. In 2021, the USAID-funded Digital Square’s COVID-19 Map & Match project conducted landscape assessments that identified global goods already in use in 23 countries (one of which, Burkina Faso, is included in this study’s country selection) [10]. While some information exists about which global goods were adapted for COVID-19 vaccination management, there are few studies providing LMIC-specific context on the adoption, adaptation and scaling of global goods for countries’ COVID-19 surveillance and vaccine response [11].

Starting in 2022, the USAID-funded Country Health Information Systems and Data Use (CHISU) program supported selected countries to improve their COVID-19 data management and use. In the three study countries—Burkina Faso, Mali and Suriname—ministries of health and their partners managed each country’s COVID-19 rapid response and created vertical or separate surveillance or vaccine management information systems that operated in parallel to the existing national HMIS. CHISU supported each country to adopt or customize DHIS2 to manage the COVID-19 vaccination response.

Table 1 demonstrates the COVID-19 vaccination context in each of the three study countries, informed by available WHO data.

**Table 1:** COVID-19 vaccination country contexts [12]

	Burkina Faso	Mali	Suriname
Timeline	June 2021–March 2023	March 2021–May 2023	February 2021–June 2023
Vaccination doses delivered	6.7 million	5.9 million	555 000
Doses per 100 population	32	29	95

### Country context

Burkina Faso has used global goods software for health data since 2013, when it established its DHIS2-based national routine HMIS called Entrepôt National des Données de la Santé (ENDOS) [13]. In 2019, USAID funded MEASURE Evaluation to support the country’s ministry of health (MOH) in establishing a One Health information system to create a comprehensive and effective response to zoonotic and key human health threats. The One Health system, developed on the DHIS2 platform, encompasses disease surveillance across human, animal and environmental health information systems, laboratory networks and emergency responses. One instance within the One Health information system is the MOH’s disease surveillance database, called MS-Surveillance, which collects aggregate and individual-level data [14]. CHISU’s implementation experience found that other uses of DHIS2 in Burkina Faso outside of the COVID-19 pandemic and associated investments include a custom DHIS2 instance for logistics management called NetSIGL 2.0, established in March 2022, and Tracker modules established for the management of tuberculosis data in 2019 and HIV data in 2020. Other global goods software used in Burkina Faso include CommCare and RapidPro for community health data management.

Mali’s MOH established DHIS2 as its HMIS in 2016. The HMIS includes aggregate and individual-level disease surveillance data [15]. In 2018, Mali adopted and customized Tracker for individual-level data management for pregnant women and child immunization. In 2019, Tracker applications were configured for management of patient-level data for HIV, tuberculosis, community health workers and maternal and child health. CommCare is another global good that was introduced in Mali in 2017 by Terre des Hommes and has been used since to strengthen the integrated management of childhood illness through the digitization of medical protocols (care algorithms), quality improvement and continuous training through e-learning.

There is limited publicly available information about Suriname’s digital health ecosystem. When the pandemic arrived, Suriname was beginning to build awareness about and lay the foundation for its digital transformation [16]. Prior to COVID-19’s arrival, the national HIS was primarily paper-based. The 2021 Global Health Security Index reported that there was no public evidence that electronic health records were commonly used or that data standards for electronic health data existed in Suriname [17]. CHISU’s implementation experience found that while some digitized systems existed, the use of digital global goods has been limited in the country. For example, Suriname’s MOH began piloting DHIS2 (aggregate and Tracker) for malaria in July 2020, but the system had not yet been scaled to the national level at the time of this study. In 2023, the Information Systems for Health (IS4H) initiative and Suriname’s MOH published an Agenda for the Digital Transformation of the Health Sector in Suriname [18].

### Study aims

This study explored how and why stakeholders selected global goods software to use in their country’s COVID-19 vaccine response efforts, challenges encountered and lessons learned from the experience. One aim of the study was to help governments, donors and partners understand key leverage points for HIS strengthening, particularly in emergency contexts. The findings seek to provide insights into how effective digital health interventions were realized and what challenges arose that should be considered in determining future digital health investments. Additionally, this study aimed to provide country-specific lessons learned around the process of selecting, using and adapting global goods for countries’ COVID-19 vaccination response to benefit governments, donors and stakeholders in planning their investments in global goods for future health emergency responses.

## MATERIALS AND METHODS

### Study design

We designed a qualitative study as part of USAID’s COVID-19 Digital Collaborative Learning Agenda (henceforth ‘Learning Agenda’). We used one of the four Learning Agenda questions as the guiding question for this study:

To what extent and how did USAID’s COVID-19 health system, data and digital health investments leverage existing or develop new global goods, resulting in standard operational, functional and system requirements that advance country replication and/or increase global digital and data system investment repositories? [1]

Our research questions related to the guiding question were:

1. How were specific global goods selected, used and/or adapted for the COVID-19 vaccination response in selected countries?
2. Why were specific global goods used and/or adapted for the COVID-19 vaccination response in selected countries?
3. What lessons learned were gathered from selecting, using and/or adapting global goods for the COVID-19 vaccination response in selected countries?

#### Analytical framework

The Learning Agenda’s theory of change served as an analytical framework that informed research questions and the design of study data collection instruments [1, Figure 1]. The theory of change mapped the relationship between USAID’s COVID-19 vaccination data and digital investments on immediate outcomes related to the vaccination effort—as well as potential longer-term outcomes related to strengthening each country’s digital health enabling environment and health system.

With this study, we intended to explore and document, where relevant, the pathway between interventions (guidance materials; architecture and integration; global goods; data capture and planning tools; training, coaching and supportive supervision; and data quality interventions) and the immediate outcomes related to data collection, information systems and processes, and data quality, analysis and use at the country level. We also hoped to explore and document, where relevant, a pathway to the intermediate outcomes of supporting COVID-19 vaccination goals and improved use of data, with some initial indications that specific priority populations have been reached, as well as a pathway to strengthened digital health enabling environment.

We utilized the theory of change under the assumption that we would not substantiate pathways from interventions to outcomes that fell outside the scope of CHISU implementation activities, and that it would not be feasible within the timeframe of the learning activity to substantiate pathways to long-term outcomes and impacts that can be categorized by WHO and the International Telecommunication Union (WHO-ITU) eHealth building blocks [1,19]. We used the HIS Stages of Continuous Improvement measurement framework and the digital health global goods maturity model to inform the development of the data collection instruments to elicit information on the extent of adaptation of digital global goods for the COVID-19 response [20, 21].

#### Study setting and sampling criteria

The study was implemented between January and June 2023, with data collection carried out between March and May 2023. The research team comprised CHISU monitoring, evaluation and learning staff and resident advisors in the study countries, who represented a mix of backgrounds and skills relevant to digital health and qualitative research.

The research team identified five eligible countries where CHISU received COVID-19 funding between 2022 and 2023 for activities that included support to the MOH for COVID-19 data management and use involving any global goods software. We selected Burkina Faso, Mali and Suriname for inclusion in the study because they met the eligibility criteria and we received the required approvals to conduct data collection within the Learning Agenda timeframe. Ghana and Haiti were eligible but did not participate. While use of DHIS2 for COVID-19 vaccine data management was not a factor in the country sampling criteria, all countries selected were using the software.

The research team identified 29 different types of respondents, including individuals from national- and regional-level government units, donors and implementing partners, across the study countries. We then used purposive sampling to identify specific individuals from those bodies to invite to participate in key informant interviews (KIIs) if they met the following eligibility criteria:

(1) Managed and/or led COVID-19-related HIS activities at the national or subnational level, with significant involvement in COVID-19 data management and/or vaccination campaignsOR
(2) Worked for a technical and/or financial partner who funded or provided technical assistance to the national COVID-19 responseAND
(3) Were at least 18 years of age.

### Data collection and analysis

#### Data collection

We developed a semi-structured interview guide for KIIs based on our research questions and analytical frameworks. The guide was prepared in English, validated by CHISU resident advisors for country context, then translated into French. The guide was adapted within the individual country contexts during implementation based on emerging insights.

The research team primarily held interviews in person; three took place virtually over Zoom to accommodate respondent schedules and one took place in a hybrid format with respondents on Zoom and in person. One interviewer led the interviews and one notetaker participated. KIIs were conducted in French in Burkina Faso and Mali and in English in Suriname. KIIs lasted from 30 minutes to 2 hours. Each KII was audio-recorded and transcribed verbatim in the original language. For analysis, French transcripts were translated to English using DeepL software with staff review. The research teams took notes during KIIs and wrote debrief notes after each KII. Teams met daily, either online or in person, when KIIs were carried out.

#### Data analysis

Identifying information for KII respondents was removed from the KII transcripts prior to analysis. We analyzed the interview data using Dedoose, a qualitative analysis software. The research team developed a codebook of deductive descriptive codes that summarized the main topic of each coded section of the transcript. Five main deductive codes (parent codes) and eight deductive subcodes (child codes) were created in Dedoose for descriptive coding. Inductive descriptive codes were created when a new topic not previously included in the codebook was identified in the data. When coding was completed, descriptive codes were tabulated to identify patterns in the data and relationships among different codes.

## RESULTS

We interviewed 36 individuals across the three selected countries: 15 respondents across 12 KIIs in Burkina Faso; 12 respondents across 10 KIIs in Mali; and nine respondents across six KIIs in Suriname. These individuals came from 27 different agencies or organizations, including 17 of the 29 initially targeted types of respondents. When respondents from the targeted groups were not available and the research team identified additional eligible individuals, they were invited to participate. Respondents represented 16 government departments or agencies and 11 other technical and financial partners (TFPs) across the study countries. Table 2 provides a summary of the interview respondents by category and country.

The results from these KIIs are presented below, organized by the three research questions to describe the process of DHIS2 selection and adaptation, factors that influenced the use of DHIS2 and lessons learned from the selection and implementation of DHIS2 for COVID-19 vaccination response.

**Table 2:** Interview respondents

Respondent Category	Burkina Faso	Mali	Suriname	Total
Total number of respondents	15	12	9	36
Total number of interviews conducted	12	10	6	28
Total number of agencies and organizations	12	9	6	27
Government agency, directorate, office or unit	9	4	3	16
Technical or financial partner organization	3	5	3	11

### DHIS2 selection and adaptation process for COVID-19 vaccination response

In this section, we highlight the process by which each of the study countries adopted and customized DHIS2 for COVID-19 vaccine management and surveillance data. The subsections, organized by study country, describe elements of the country-specific selection and adaptation processes, using insights gathered from the KIIs.

#### Burkina Faso

Beginning in 2020 in Burkina Faso, MS-Surveillance was used for COVID-19 surveillance—tracking and reporting cases, tests and deaths—using Tracker for individual data. Burkina Faso’s MOH led (and many other partners were involved in) the decision to adopt DHIS2.^1^ A respondent shared that Burkina Faso was one of the first countries to set up its COVID-19 testing module in Tracker in April 2020 and presented the module at the DHIS2 Conference to share its experience with other countries: ‘The Ministry had the resources in-house to do it … They were able to get their DHIS2 Trackers out before the University of Oslo proposed a Tracker’.

Respondents shared that as soon as the first vaccines arrived in Burkina Faso in June 2021, the MOH and its partners rapidly developed paper and digital tools for collecting COVID-19 vaccination data to enable more efficient data feedback and management at all levels of the health system. Multiple respondents stated that the MOH considered several electronic data management tools for COVID-19 vaccination data, including CommCare. The MOH decided to use a DHIS2 aggregate system for COVID-19 vaccine data management after considering factors such as local hosting, government ownership of the platform and availability of partner support. Along with its partners, the MOH customized its Tracker for COVID-19 vaccination data collection using the WHO-disseminated metadata package. They first used this system in June 2021. Respondents mentioned a mix of funding for the development and improvement of the COVID-19 vaccination management tools, including funds from USAID, the Global Fund and the Bill & Melinda Gates Foundation. Respondents did not indicate that donor preference was a factor in the selection of DHIS2, but availability of TFP assistance to adapt the platform to the ministry’s needs was an important consideration.

#### Mali

Respondents in Mali described that two entities within the MOH’s General Directorate of Health and Public Hygiene led different aspects of the COVID-19 pandemic response and associated data flows: the Epidemiological Surveillance Division managed surveillance while the National Immunization Center managed vaccination. The MOH first established a COVID-19 data management system in Microsoft Excel. When the WHO standard surveillance package for DHIS2 became available in 2020, the Epidemiological Surveillance Division and TFPs adapted it to the Malian context.^2^ Respondents shared that Tracker forms were configured for case management and testing. In June 2021, the National Immunization Center and partners configured a Tracker form to manage COVID-19 vaccination data using the WHO-disseminated metadata package. Stakeholders considered and valued the fact that DHIS2 is an open-source, mature global good. One respondent stated, ‘DHIS2 is already an open-source software package that’s proven its worth, having already been used in several countries. So there’s maturity in this respect [and] it’s also a tool that’s easy to use’.

A team of technical experts led the configuration of the COVID-19 systems. As described by one respondent, ‘The development process was carried out through a participatory workshop, which brought together all the key players and partners. All those involved in the health information system and immunization validated these tools’. Respondents reported that the COVID-19 Tracker forms and aggregate data are consolidated in one DHIS2 instance. The COVID-19 DHIS2 instance is separate from the HMIS and other disease surveillance DHIS2 instances, meaning that interoperability is needed to enable data exchange between the instances. Within the COVID-19 DHIS2 instance, surveillance and vaccination data are captured in separate forms and were not initially linked. According to respondents, this increased data entry time and workload because data for an individual needed to be entered separately into each system rather than once with shared basic information. Respondents described improvements in functionality that were made over time, including coordination between the MOH and partners in 2023 to link the forms within these systems, allowing for tracking one client across COVID-19 testing, case notification and vaccination. In discussing updates to the COVID-19 DHIS2 instance, a respondent said, ‘We’ve updated it constantly’.

#### Suriname

In Suriname, key actors involved in decision-making related to COVID-19 data management systems included the Bureau for Public Health, the National Immunization Program and the Pan American Health Organization (PAHO). Respondents stated that surveillance data was captured in Excel and aggregated in a Google Sheets system at the start of the pandemic in March 2020. To support the increasing need for high-quality data to track the pandemic, donors and the private sector supported the development of an online application called COVID Database using proprietary technology. Starting in June 2020, this system was used to register vitals, test results and contact tracing.

Respondents noted that when COVID-19 vaccines became available in Suriname in 2021, vaccination data was collected on paper and these records were subsequently manually transferred to Excel, then aggregated in Google Sheets. Respondents shared that the MOH considered implementing a vaccination registry as a new functionality within the COVID Database, but it proved to be costly and unsustainable.

According to respondents, the MOH decided to use DHIS2 for the COVID-19 vaccine registry in May 2021, after receiving technical and financial support from PAHO. Initially, DHIS2 aggregate was customized using the DHIS2 COVID-19 Vaccine Delivery Toolkit metadata package designed to align with WHO guidance on national deployment and vaccination plan data standards. The MOH began using Tracker exclusively for COVID-19 vaccine data in July 2021. To ensure the new digital system was comprehensive, teams performed intensive data cleaning on records previously collected on paper before importing them into DHIS2.

There were some delays in establishing DHIS2 for COVID-19 data management once it was selected. One respondent stated: ‘The initial discussions of the DHIS2 took a lot of time. Where would the server be, would it be outside of [the country] … the system was ready and the DHIS2 module for vaccination was there … but these kinds of decisions took a lot of time’. Once those decisions were made, there were extensive data entry efforts during the transition from paper-based tools to DHIS2. Hundreds of Excel files were merged before import into DHIS2 and vaccine records were reviewed and checked for errors. One respondent said, ‘We had all this data on paper, hard copy, for people to enter into DHIS2, and that was frustrating’. Respondents noted that, at the time of the interviews, there were still backlogged data and ongoing data cleaning needs.

### Factors influencing the use of DHIS2 for COVID-19 vaccination response

Three themes emerged relating to the factors primarily responsible for why DHIS2 was selected for COVID-19 vaccination response in the study countries: familiarity with the tool, urgent demand for data and partner influence. The following subsections present findings organized by these areas.

#### Familiarity with the tool encouraged countries to leverage existing global goods systems

In Mali and Burkina Faso, prior experience using DHIS2 for health data influenced the MOH decision to implement the system for COVID-19 data management. Because MS-Surveillance was already established on the DHIS2 platform in Burkina Faso, when COVID-19 arrived, a respondent noted, ‘All we had to do was strengthen it so that it could now take into account COVID vaccination. We didn’t have to reinvent a new system to collect and manage vaccination data’. Familiarity with the platform meant that there would not be a significant training burden: ‘A lot of the ministry’s staff have already been trained in the use of DHIS2 … which makes it easier to use. I guess that’s why DHIS2 was chosen for the vaccine tracking’.

Burkina Faso considered utilizing CommCare as its COVID-19 data management system, but had a broader existing experience using DHIS2 for disease surveillance. One respondent stated:

‘We said: when we have the One Health platform, why not take advantage of that to develop the COVID-19 platform for data management, such as notification, case follow-up, contact cards … point-of-entry monitoring, COVID results and all. So that’s what motivated the implementation of the COVID-19 management system at the level of the One Health platform that we call MS-Surveillance’.

In Mali, DHIS2 was selected as the electronic COVID-19 data management system largely due to the existing country capacity and familiarity with the platform. One respondent in Mali stated:

‘When COVID arrived, there was already a system in place … This is what led to the idea of creating an application in DHIS2 called COVID surveillance, and when vaccination came along, they created the vaccination model because they knew that global system, people were trained and people were already comfortable using DHIS2, so they went with that’.

Despite some challenges with adapting DHIS2 as the sole COVID-19 data management tool, stakeholders in Mali were familiar with using DHIS2. One respondent stated:

‘The national strategy of the health information system is the use of DHIS2 at all levels, from the central level to the community level. Everyone uses DHIS2 as a source for reporting immunization data, whether it is routine or not. Since 2016 Malians have been using this platform, and people have been trained, equipped with computers, tablets and all necessary equipment’.

#### Data demand created an urgency to quickly establish COVID-19 data systems

Across the three countries, respondents cited the need for timely data as a factor in the COVID-19 vaccine information system decision-making process. In each country, this resulted in the use of both Excel-based tools and DHIS2. A respondent from Burkina Faso stated: ‘At the start there was a demand to go fairly quickly, given the urgency and the context’. Excel tools offered one solution to a rapid data management system because of the ease of entering aggregate data. One respondent in Burkina Faso stated, ‘Information in DHIS2 is often individual, it takes time, it goes up very slowly. People need information right away, so we created more simplified tools that allow us to get the information quickly through Excel’. Respondents also highlighted that Excel-based tools were used for aggregate data synthesis and visualization because of initial data quality issues with Tracker, including delays or gaps in data entry for COVID-19 cases and vaccination caused by a lack of human resources. This resulted in discrepancies between what was considered the official data reported in Excel files and those in MS-Surveillance. However, respondents noted that sharing and standardization of Excel files were challenges that contributed to support for the use of Tracker, which accommodated more complex analysis.

In Mali, there was an early-pandemic proliferation of systems to meet competing demands and requests from various stakeholders. Throughout the pandemic, both DHIS2 and Excel were used for COVID-19 vaccination data. While some of the DHIS2 adaptations in Mali focused on aligning the digital system with Excel sheets, the parallel structure resulted in ongoing data quality issues and disparities between data in different systems. One respondent stated, ‘You see parallel tools because following projects or programs, there are indicators that a partner requires with the funding agreement, so you have information or indicators that perhaps aren’t in DHIS2 that you’re obliged to provide’.

A respondent in Mali shared that while daily COVID-19 case dashboards are shared by the MOH using aggregate data from Excel, ‘We’d like it to be automatic in the [DHIS2] system, so that this information can be checked by everyone who has access to the system’. Respondents noted that aggregate data collection in Excel has lasted longer than envisioned and that many TFPs have supported transition plans to move COVID-19 data entirely to DHIS2 by 2024. One respondent explained that it has been challenging to transition away from Excel: ‘We’re facing certain difficulties with the leadership, the vaccination team, abandoning the Excel file. Because they’re used to Excel. DHIS2 is there … but right now they’re saying we need to make a transition plan’. Excel is viewed as a trusted tool for some decision-makers, in part due to the data demands from donors as well as concerns around availability of equipment, internet connectivity and training for DHIS2 to be used effectively. One respondent stated:

‘Even with aggregated data [in DHIS2], we still had trouble getting real-time data. We’ve had a number of discussions here about the fact that the data isn’t in real time, whereas USAID wants us to give them the information every two weeks, i.e. how many vaccinations have been carried out in each zone … So, we often have to create parallel files to get the information … Because if we don’t have any information [in DHIS2], we're obliged to … develop other files so that we can get the information quickly’.

Despite Excel being viewed as a useful tool, using DHIS2 allowed for the centralization of data on a server.

Suriname also started using Excel for COVID-19 data management, then implemented the custom system known as COVID Database before deciding to use DHIS2. A respondent explained why the MOH initially chose Excel: ‘A lot of people are familiar with Excel, and you would have to train everyone who will enter data … in DHIS2’. Widespread experience with Excel among the health workforce involved in COVID-19 vaccination data management was cited as the primary reason it was the first tool selected. However, it was not viewed as a permanent solution. Another respondent explained why the custom COVID Database system was not sustained: ‘When it was almost ready to use, it was presented to the ministry and it was not a system that was … interoperable’. Because the MOH had already begun planning for a national digital health architecture that eventually will include a health information exchange for most data systems in the Surinamese healthcare system, DHIS2 was chosen for its interoperability potential with the future health information exchange.

#### Partners influenced each MOH’s decision to use DHIS2

TFPs’ support of and technical experience with using DHIS2 played a role in each study country’s decision to use DHIS2. In Burkina Faso, a respondent described that for the customization of the DHIS2 COVID-19 vaccination tools, ‘there was a consistent mobilization of partners around who were keen to understand what the ministry’s needs were, and who agreed to finance these needs and really provide the technical and financial assistance to make these things happen’.

In Mali, respondents indicated that while the MOH selected DHIS2, the availability of TFPs to fund, customize and adapt the tool was critical for its implementation. Partner collaboration contributed to the improvement of the DHIS2 COVID-19 vaccination tools over time, yet was not without challenges. When additional system customization was needed, the MOH and TFPs engaged in a collaborative process through which they agreed on the data elements and reached a consensus. TFPs hosted workshops and data quality review meetings to harmonize the Excel and DHIS2 COVID-19 data systems and to validate the data. Respondents noted challenges in collaboration, as data was not always shared across all stakeholders. The division of responsibilities between partners involved in COVID-19 surveillance and vaccination was viewed as an area that could be improved moving forward. One respondent in Mali stated: ‘Each partner, at some point, wants to show that they can do it better. And the country has goals too. It’s about setting up a strong coordination system from the outset so that partners can talk to each other and there’s a clear direction as to who can do what; I think this coordination could be better structured in relation to vaccination’.

Advocacy from PAHO in favor of the adoption of DHIS2 played a significant role in its selection in Suriname. PAHO financed its development through technical assistance from the University of Oslo. A respondent stated, ‘We did not have the finance to have another system developed’. The respondent described that PAHO invested in the initial customization of DHIS2 and currently supports the digital transformation of the health sector in Suriname, which includes the development of the national health information exchange.

### Lessons learned from selecting, using and/or adapting DHIS2 for COVID-19 vaccination response

Three themes emerged relating to the overall lessons learned from the study countries’ experiences selecting, using or adapting DHIS2 for COVID-19 data management. The following subsections present the findings from the KIIs organized by the role of supportive supervision and reinforcement of data quality, contextual factors in each country and the envisioned expansion of DHIS2 for other health areas.

#### Supportive supervision and reinforcement of data quality were seen as vital

Respondents in all three countries noted that measures taken to support historical data entry, data quality review and supportive supervision were critical to the effective use of DHIS2 for COVID-19 data management. This was particularly evident through the use of both paper-based and electronic systems and the ongoing efforts to address continued data disparities between them. A respondent in Suriname stated: ‘There are still some data that need to be checked within the [DHIS2] system because … not all the data are being entered correctly’. A respondent in Burkina Faso stated: ‘We need supervision, support and guidance from the field to give them the tools they need to make the system work the way we want it to. It works, but with shortcomings because there are actors who are poorly equipped, who are not trained, who do not have the necessary skills’.

At the time of data collection, respondents in Mali expressed ongoing concerns about data quality (completeness, timeliness and data entry errors) in Tracker and continued disparities between aggregate and Tracker data. Some respondents attributed this to the health workers’ heavy workload, the lack of personnel dedicated to data entry and the amount of time required for entering individual-level data. Over time, some forms were streamlined and others incorporated new data elements to meet data demands. However, when Excel tools were revised, the changes made to forms were not always updated in DHIS2, leading to discrepancies.

Regular data quality review meetings were key components of improving COVID-19 vaccine data quality for decision-making in Burkina Faso and Mali. A respondent in Mali said, ‘Since the start of the 2021 vaccination campaign, we’ve been holding meetings twice a week, on Tuesdays and Fridays, for ~2 hours, where the ministry’s executives, all the partners involved in managing vaccination data and the regions are connected to share the results in real time in order to see what planning is still to come’. The meetings allow stakeholders to discuss obstacles to improving data quality and how to address them. Another respondent in Mali stated that supportive supervision allows managers ‘to see the difficulty that people have with regard to data entry, with regard to analysis. And there have been a lot of follow-up missions, online user orientation sessions, physical trainings, data reviews and formal supervisions … so all of that has made the data easier to use’.

#### Contextual factors presented challenges in the implementation of DHIS2 for COVID-19 vaccine and surveillance data management

Contextual factors such as coordination across actors, alignment on priorities, funding availability and sufficient electricity and internet connectivity presented some challenges in the implementation of DHIS2 for COVID-19 data management. Respondents in Burkina Faso cited challenges related to the use of DHIS2 for COVID-19 vaccine management, including lack of reliable electricity and internet connectivity in some areas of the country, concerns about server capacity, the need for additional training of system users, financial constraints relating to version upgrades and the burden of individual data collection in the case of Tracker. Respondents also discussed coordination and collaboration across actors as both a strength and a challenge in terms of the configuration and use of the DHIS2 COVID-19 vaccine management systems. One respondent noted that while generally each ministry operates separately in their one area of expertise, COVID-19 encouraged more collaboration. Thematic working groups, established based on stakeholder areas of expertise, allowed for better pandemic response decision-making. However, information was not always collected or shared with all partners in a standardized way. One respondent in Burkina Faso expressed interest in a more widely available DHIS2 dashboard so that COVID-19 vaccination efforts could be more visible to partners. Different stakeholders’ interests in different data elements also led to conflicting requests regarding modification of data collection tools. A respondent stated, ‘We need to … get the players to agree on the data collection method, and above all there should be much greater clarity at statutory level in terms of responsibility’.

Respondents in Mali noted that the main challenges in the use of DHIS2 were related to internet connectivity, power outages, funding availability and data quality issues. Lack of internet connection at the health facility level affected data entry and contributed to incomplete data. One respondent in Mali stated that while it is ideal for the government to have the budget to fund the COVID-19 data systems, ‘we are not there yet. We still call on our partners, notably USAID and the Global Fund, who have supported us’.

In Suriname, respondents mentioned the primary challenges of internet connectivity, funding availability and training needs for capacity development in the use of DHIS2. There have been ongoing challenges with the availability of data dashboards to users outside of the MOH and challenges with communications about changes in the availability of COVID-19 data to the public. At the time of this study’s data collection, multiple respondents said that official information was last available in 2022 because the MOH had stopped sharing COVID-19 data publicly.

#### Envisioned expansion of global goods based on COVID-19 experiences

Respondents in each of the countries referred to current or future plans to implement Tracker for routine immunization services or other health areas based on their experiences using DHIS2 for COVID-19 vaccine management and other areas of the COVID-19 response. Respondents in Burkina Faso discussed the expanded use of Tracker as a major outcome of the COVID-19 response. Respondents noted that before COVID-19, DHIS2 was used primarily for aggregate data, not individual data. Through the process of developing and adapting COVID-19 Tracker, the MOH and partners now have increased familiarity and capacity to use the Tracker application. Respondents noted that since the start of the COVID-19 pandemic, Burkina Faso has developed additional Tracker forms for other health areas, including cervical cancer, distribution management of long-lasting insecticidal mosquito nets and seasonal malaria chemoprevention.

Though Mali had experience using the Tracker application before the COVID-19 pandemic, additional training and supervision were required for the use of Tracker rather than DHIS2 aggregate. A respondent in Mali stated, ‘The vaccinators first have to be trained on how to enter individual data. For aggregate data, the agents are already trained, so there’s no problem. They can just do a little orientation and it’s done. But for individual data, it’s the vaccinators who need to have tablets to do the data entry’. Despite the increased training burden, Tracker was viewed as a valuable tool that helped to resolve some reporting challenges encountered with DHIS2 aggregate. One respondent shared, ‘The majority of measures that were not taken into account by the aggregate have been taken into account by Tracker’. Another respondent noted that the experience using DHIS2 for COVID-19 data management has sparked partner conversations about digitizing community health data in Mali.

Respondents in Suriname shared their views that the use of Tracker for COVID-19 vaccination data has been largely successful. While it was designed to be used specifically for COVID-19 vaccination data, there are now discussions about configuring the system to encompass all vaccination data. A respondent stated, ‘We are thinking of including COVID vaccinations into the routine vaccines’. PAHO is currently supporting Suriname’s MOH to develop and implement another Tracker to register adverse events following immunization.

## DISCUSSION

### Selection and adaptation process

This study found that the process through which the MOH reached the point of implementing DHIS2 for COVID-19 vaccination data management looked different in each country due to the distinct COVID-19 contexts and timelines, partner and donor influence and involvement and digital tools that were already in use. The divergent experiences respondents shared converged around each MOH’s decision to use DHIS2 for COVID-19 data management.

TFPs supported MOHs in each of the three countries in the decision to use DHIS2 for COVID-19 vaccination data management, and were especially important as MOHs customized and implemented Tracker. WHO’s endorsement of the DHIS2 COVID-19 metadata package was a potential influencing factor for the establishment and/or expansion of DHIS2 instances for COVID-19 data management in Burkina Faso, Mali and Suriname, as discussed further in the following subsection.

### Influencing factors

Based on the experiences respondents in each country shared, the research team identified three key factors that most influenced the use of DHIS2 aggregate and Tracker applications in all three study countries: familiarity with the tool, data demand and partner influence. Prior to the COVID-19 pandemic, Burkina Faso and Mali already used DHIS2 as their national HMIS. Suriname did not but had piloted DHIS2 for malaria programs. This study found that in countries where DHIS2 was already widely in use as the national HMIS, and that had received prior investments focused on routine health services, such as Burkina Faso and Mali, there was a respondent perception of a lighter burden during the emergency response in terms of customizing new forms and dashboards and training end-users. These countries leveraged the use of DHIS2 for COVID-19 vaccination data management. In Suriname, where DHIS2 was not used previously apart from a malaria instance, the global-level investment in DHIS2 to share metadata packages and other technical resources supported Suriname’s adoption of the tool during the emergency response. In this newer setting, this study found that a larger need remains for training and building in-country capacity to expand and sustain the use of the global good and provide access to using the data.

### Lessons learned

This study found three main categories of lessons learned from Burkina Faso, Mali and Suriname’s experiences selecting, customizing and utilizing DHIS2 for COVID-19 vaccine data management. First, supportive supervision and reinforcement of data quality were key factors in the effective use of DHIS2. Second, contextual factors including coordination across actors, funding availability, adequate infrastructure and alignment on priorities posed some challenges to the countries’ implementation of DHIS2 for COVID-19 vaccination data management. Third, experience using the tool throughout the pandemic led to plans for expanding the use of Tracker for routine immunization or other health areas. While individual data entry on Tracker was described as increasing data entry time and requiring additional resources for training, respondents noted that it ultimately allowed for more in-depth analysis. This additional functionality contributed to the interest in expanding the use of DHIS2 beyond COVID-19 data.

### Theory of change substantiation

Through our findings, we saw evidence to strengthen the broader digital health enabling environment (as illustrated in the Learning Agenda’s theory of change) through increased services and applications for COVID-19 data management and the use of Tracker forms for other health areas. Furthermore, this and other work supported by USAID toward the use and expansion of global goods are likely to contribute in the near future to the WHO-ITU eHealth building blocks of ‘leadership and governance’ and ‘standards and interoperability’ [19].

### Limitations

This study had several limitations. Purposive sampling was used for the interviews, which enabled us to identify respondents based on their close involvement with the study topics of interest and which we believe mitigates potential limitations of the small sample size. This introduced a level of selection bias in the study population. While the study aimed to answer the question of how specific global goods were selected and adapted for COVID-19 data management, our findings did not speak to some components that may have been part of the decision-making process, including requirements gathering. The findings of this research are context-specific but the experiences shared may be relevant to other country contexts.

## CONCLUSION

### Utilize familiar global goods during health emergencies

Global goods, including DHIS2, provided an important, relatively low-burden option for standardized, adaptable data systems in an emergency response—both in countries with extensive experience using it (Burkina Faso and Mali) and in a country with relatively limited experience (Suriname). While the use of DHIS2 for COVID-19 data management had advantages in these country contexts, it is possible that other digital global goods or other systems could also have been well-suited for COVID-19 data management. In Mali, Excel tools are still being used in parallel to DHIS2, and in all three countries, there are data completeness and quality concerns. Ultimately, the demand for COVID-19 data drove these countries’ selection and development of their DHIS2 instances. The availability of a digital global good that was already established in the country and where there was capacity (either in-country or through TFPs) supported the adoption and development of DHIS2 for COVID-19 data management. Therefore, we recommend that MOHs consider utilizing familiar global goods during health emergency periods to minimize the financial and management burdens of establishing a new information system.

### Coordinate funding for streamlined digital system investments

With global goods systems such as DHIS2 now in place in countries including Burkina Faso, Mali and Suriname, significant continued investment is needed to maintain them and support the expanded use of individual data. Investments made now may reduce the burden of customizing and scaling similar systems in future health emergency response contexts, but competing donor data demands may contribute to the continued proliferation of parallel systems. Therefore, we encourage close donor coordination and alignment to streamline digital system investments to help prevent digital system proliferation and conflicting data demands in future health emergency contexts.

### Invest in bolstering contextual factors as interventions

The study results demonstrate that investments are often needed to bolster areas that were included in the theory of change as contextual factors, such as external support to coordinate across multiple actors, funding availability for interventions and adequate infrastructure. These factors cannot be assumed to exist at a level that provides the necessary enabling environment for global goods solutions to operate effectively. Stakeholder alignment on priorities can promote timely decisions on issues including global goods selection, server expansion and interoperability. Sufficient electricity and internet connectivity was a key area of external investment in all three of the study countries. The status of these elements at the outset of and throughout the pandemic required consistent investment and was crucial to the feasibility of the success and sustainability of the digital health interventions. We recommend that TFPs support countries to invest in the enabling environment for global goods systems. These investments will allow countries to operate effectively by assessing these contextual factor areas, providing targeted inputs to bolster the areas where gaps are identified and supporting sustainability planning.

## Data Availability

The data underlying this article will be shared upon reasonable request to the corresponding author.

## Footnotes

1 Stakeholders mentioned in the interviews included: Blue Square, Data for Implementation, Digital Square, CHISU, Global Fund, Living Goods, UNICEF, USAID, WHO and government agencies, including the National Agency for the Promotion of ICT, Department of the Protection of Population Health, Department of Prevention through Vaccination, Department of Health Information Systems, Expanded Program on Immunization, Executive Secretary of One Health, Health Emergency Response Operations Center, Health Information and Epidemiological Surveillance Centers, General Department of Studies and Sector Statistics and regional health departments.

2 Stakeholders mentioned in the interviews included: National Immunization Center, General Directorate of Health and Public Hygiene, National Agency for Telehealth and Medical Informatics, National Institute of Public Health, Epidemiological Surveillance Division, Planning and Statistics Unit and TFPs including CHISU, Catholic Relief Services, Digital Square, FHI 360, HISP, Keneya Sinsi Wale, MOMENTUM Integrated Health Resilience, MOMENTUM Routine Immunization Transformation and Equity, PATH, PSI, USAID, World Bank and WHO.
